# Supplementary material for: 13C tracing in synaptosomes reveals that SGLT2 inhibition with dapagliflozin prevents metabolic deficits in the 5X-FAD model of Alzheimer’s Disease
Source: bioRxiv. 2025 May 3:2025.04.30.651373. Preprint. [Version 1] doi: 10.1101/2025.04.30.651373 (PMC12247946; doi:10.1101/2025.04.30.651373)
Supplement: Supplement 1 [file media-1.pdf]

Supplemental Table 1: Metabolite abundances in cortical synaptosomes acutely offered glucose and pyruvate

	BOTH SEXES (nmol/mg protein)			
	WT		5X-FAD Control	
	Control	DAPA	Control	DAPA
**ALANINE**	**0.83**	**0.83**	**0.99**	**0.94**
S.E.M.	0.09	0.06	0.10	0.07
**ASPARTATE**	**12.30**	**13.69**	**9.84**	**14.86**
S.E.M.	2.43	2.52	2.20	3.14
**CITRATE**	**0.43**	**0.68**	**0.38**	**0.66**
S.E.M.	0.10	0.13	0.09	0.12
**FUMARATE**	**0.87**	**0.98**	**0.80**	**0.84**
S.E.M.	0.17	0.16	0.17	0.13
**GLUTAMATE**	**16.66**	**19.57**	**17.81**	**19.50**
S.E.M.	2.14	2.77	2.16	1.75
**LACTATE**	**15.19**	**14.22**	**14.04**	**13.94**
S.E.M.	2.21	2.01	2.44	2.75
**MALATE**	**3.83**	**4.68**	**3.12**	**4.24**
S.E.M.	0.85	0.77	0.85	0.74
**PYRUVATE**	**2.95**	**3.31**	**3.25**	**2.94**
S.E.M.	0.41	0.66	0.51	0.42
**SERINE**	**0.79**	**0.71**	**0.63**	**0.91**
S.E.M.	0.17	0.11	0.08	0.12
**SUCCINATE**	**0.99**	**1.00**	**0.89**	**1.22**
S.E.M.	0.15	0.12	0.15	0.19

	FEMALE (nmol/mg protein)			
	WT		5X-FAD Control	
	Control	DAPA	Control	DAPA
**ALANINE**	**0.75**	**0.74**	**0.94**	**0.93**
S.E.M.	0.10	0.11	0.17	0.08
**ASPARTATE**	**7.82**	**7.00**	**5.28**	**7.58**
S.E.M.	1.52	1.26	0.77	0.59
**CITRATE**	**0.27**	**0.30**	**0.18**	**0.38 ***
S.E.M.	0.05	0.04	0.01	0.04
**FUMARATE**	**0.57**	**0.49**	**0.45**	**0.58**
S.E.M.	0.12	0.09	0.14	0.11
**GLUTAMATE**	**14.76**	**14.71**	**12.64**	**16.35**
S.E.M.	3.10	3.26	1.78	0.72
**LACTATE**	**15.86**	**14.99**	**17.91**	**20.72**
S.E.M.	1.94	2.70	3.29	2.68
**MALATE**	**2.42**	**2.12**	**1.25**	**2.45 ***
S.E.M.	0.59	0.32	0.26	0.26
**PYRUVATE**	**2.54**	**3.37**	**3.57**	**2.86**
S.E.M.	0.51	1.39	0.98	0.61
**SERINE**	**0.47**	**0.44**	**0.52**	**0.83**
S.E.M.	0.09	0.14	0.12	0.21
**SUCCINATE**	**0.74**	**0.71**	**0.52**	**0.79**
S.E.M.	0.18	0.16	0.08	0.08

	MALE (nmol/mg protein)			
	WT		5X-FAD Control	
	Control	DAPA	Control	DAPA
**ALANINE**	**0.92**	**0.91**	**1.04**	**0.95**
S.E.M.	0.15	0.07	0.12	0.12
**ASPARTATE**	**16.79**	**19.26**	**14.40**	**22.15**
S.E.M.	3.78	2.93	2.86	4.19
**CITRATE**	**0.59**	**1.00**	**0.57**	**0.94**
S.E.M.	0.17	0.14	0.10	0.14
**FUMARATE**	**1.18**	**1.38**	**1.15**	**1.09**
S.E.M.	0.28	0.13	0.18	0.18
**GLUTAMATE**	**18.57**	**23.62**	**22.99**	**22.65**
S.E.M.	3.03	3.73	1.72	2.88
**LACTATE**	**14.53**	**13.57**	**10.17**	**7.16**
S.E.M.	4.25	3.12	2.66	1.98
**MALATE**	**5.24**	**6.81**	**4.98**	**6.04**
S.E.M.	1.54	0.33	1.00	0.89
**PYRUVATE**	**3.36**	**3.26**	**2.93**	**3.02**
S.E.M.	0.65	0.56	0.45	0.64
**SERINE**	**1.10**	**0.94**	**0.75**	**0.99**
S.E.M.	0.28	0.08	0.08	0.12
**SUCCINATE**	**1.24**	**1.24**	**1.26**	**1.66**
S.E.M.	0.20	0.12	0.06	0.25
