# Supplementary material for: 13C tracing in synaptosomes reveals that SGLT2 inhibition with dapagliflozin prevents metabolic deficits in the 5X-FAD model of Alzheimer’s Disease
Source: bioRxiv. 2025 May 3:2025.04.30.651373. Preprint. [Version 1] doi: 10.1101/2025.04.30.651373 (PMC12247946; doi:10.1101/2025.04.30.651373)
Supplement: Supplement 2 [file media-2.pdf]

Supplemental Table 2: Metabolite abundances in the retrosplenial cortex after two-month Dapagliflozin treatment.

	BOTH SEXES (nmol/mg protein)			
	WT		5X-FAD Control	
	Control	DAPA	Control	DAPA
**ALANINE**	**1.65**	**1.66**	**1.72**	**1.88**
S.E.M.	0.10	0.14	0.14	0.18
**ASPARTATE**	**5.76**	**5.11**	**5.39**	**5.79**
S.E.M.	0.40	0.41	0.38	0.64
**CITRATE**	**0.46**	**0.52**	**0.46**	**0.59**
S.E.M.	0.05	0.05	0.05	0.07
**FUMARATE**	**0.30**	**0.32**	**0.28**	**0.35**
S.E.M.	0.03	0.03	0.02	0.04
**GABA**	**3.34**	**3.57**	**3.54**	**4.11**
S.E.M.	0.35	0.39	0.41	0.50
**GLUTAMATE**	**13.69**	**14.62**	**14.53**	**15.56**
S.E.M.	0.73	1.42	1.27	1.46
**LACTATE**	**37.94**	**37.68**	**40.59**	**43.31**
S.E.M.	5.31	4.93	4.90	5.81
**MALATE**	**1.72**	**1.75**	**1.72**	**2.02**
S.E.M.	0.12	0.12	0.15	0.19
**N-acetyl aspa**	**29.17**	**28.21**	**35.12**	**32.40**
S.E.M.	5.56	4.76	10.46	8.07
**PYRUVATE**	**0.28**	**0.27**	**0.27**	**0.27**
S.E.M.	0.03	0.02	0.02	0.03
**SERINE**	**1.53**	**1.50**	**1.62**	**1.81**
S.E.M.	0.12	0.11	0.15	0.18
**SUCCINATE**	**1.24**	**1.32**	**1.44**	**1.43**
S.E.M.	0.10	0.13	0.15	0.16

	FEMALE (nmol/mg protein)			
	WT		5X-FAD Control	
	Control	DAPA	Control	DAPA
**ALANINE**	**1.59**	**1.80**	**1.65**	**1.72**
S.E.M.	0.21	0.22	0.25	0.25
**ASPARTATE**	**5.53**	**5.26**	**5.36**	**5.39**
S.E.M.	0.79	0.69	0.42	0.86
**CITRATE**	**0.40**	**0.43**	**0.42**	**0.47**
S.E.M.	0.06	0.06	0.06	0.07
**FUMARATE**	**0.28**	**0.31**	**0.26**	**0.30**
S.E.M.	0.05	0.03	0.04	0.06
**GABA**	**1.97**	**2.93**	**2.48**	**2.86**
S.E.M.	0.35	0.58	0.54	0.48
**GLUTAMATE**	**12.92**	**15.91**	**13.43**	**14.49**
S.E.M.	1.39	2.53	1.63	1.97
**LACTATE**	**54.53**	**53.71**	**54.35**	**58.23**
S.E.M.	8.95	6.79	7.89	9.17
**MALATE**	**1.79**	**1.82**	**1.59**	**1.86**
S.E.M.	0.25	0.18	0.14	0.26
**N-acetyl aspa**	**13.06**	**13.67**	**10.35**	**11.00**
S.E.M.	3.07	2.77	1.24	1.85
**PYRUVATE**	**0.28**	**0.29**	**0.28**	**0.26**
S.E.M.	0.04	0.02	0.03	0.03
**SERINE**	**1.96**	**2.12**	**1.94**	**2.09**
S.E.M.	0.25	0.33	0.22	0.28
**SUCCINATE**	**1.24**	**1.33**	**1.20**	**1.39**
S.E.M.	0.21	0.15	0.14	0.15

	MALE (nmol/mg protein)			
	WT		5X-FAD Control	
	Control	DAPA	Control	DAPA
**ALANINE**	**1.70**	**1.52**	**1.78**	**2.03**
S.E.M.	0.09	0.18	0.16	0.27
**ASPARTATE**	**5.95**	**4.96**	**5.41**	**6.15**
S.E.M.	0.39	0.47	0.63	0.96
**CITRATE**	**0.52**	**0.60**	**0.50**	**0.71**
S.E.M.	0.06	0.07	0.08	0.11
**FUMARATE**	**0.31**	**0.33**	**0.28**	**0.40**
S.E.M.	0.03	0.05	0.03	0.07
**GABA**	**4.44**	**4.20**	**4.40**	**5.24**
S.E.M.	0.20	0.46	0.47	0.68
**GLUTAMATE**	**14.30**	**13.33**	**15.43**	**16.53**
S.E.M.	0.73	1.34	1.91	2.19
**LACTATE**	**24.67**	**21.66**	**29.33**	**29.88**
S.E.M.	1.46	2.12	3.75	4.34
**MALATE**	**1.67**	**1.69**	**1.82**	**2.16**
S.E.M.	0.11	0.18	0.24	0.28
**N-acetyl aspa**	**42.07**	**42.74**	**55.39**	**51.66**
S.E.M.	7.60	6.71	16.90	12.56
**PYRUVATE**	**0.29**	**0.25**	**0.27**	**0.28**
S.E.M.	0.03	0.04	0.03	0.04
**SERINE**	**1.75**	**1.66**	**1.97**	**2.20**
S.E.M.	0.10	0.16	0.20	0.28
**SUCCINATE**	**1.24**	**1.31**	**1.63**	**1.46**
S.E.M.	0.08	0.22	0.24	0.27
